# Streptococcus pneumoniae Rapidly Translocate from the Nasopharynx through the Cribriform Plate to Invade the Outer Meninges

**DOI:** 10.1128/mbio.01024-22

**Published:** 2022-08-04

**Authors:** Teerawit Audshasai, Jonathan A. Coles, Stavros Panagiotou, Shadia Khandaker, Hannah E. Scales, Morten Kjos, Murielle Baltazar, Julie Vignau, James M. Brewer, Aras Kadioglu, Marie Yang

**Affiliations:** a Institute of Infection and Global Health, University of Liverpool, Liverpool, United Kingdom; b Faculty of Pharmacy, Mahidol University, Bangkok, Thailand; c Institute of Infection, Immunity and Inflammation, University of Glasgow, Glasgow, United Kingdom; d Faculty of Chemistry, Biotechnology and Food Science, Norwegian University of Life Sciences, Ås, Norway; e Centre de Recherche en Cancérologie et Immunologie Nantes Angers, Université de Nantes, Nantes, France; Carnegie Mellon University

**Keywords:** cribriform plate, inflammation, nose-to-meninges translocation, *Streptococcus pneumoniae*, central nervous system, infectious disease

## Abstract

The entry routes and translocation mechanisms of microorganisms or particulate materials into the central nervous system remain obscure We report here that Streptococcus pneumoniae (pneumococcus), or polystyrene microspheres of similar size, appear in the meninges of the dorsal cortex of mice within minutes of inhaled delivery. Recovery of viable bacteria from dissected tissue and fluorescence microscopy show that up to at least 72 h, pneumococci and microspheres were predominantly found in the outer of the two meninges: the pachymeninx. No pneumococci were found in blood or cerebrospinal fluid. Intravital imaging through the skull, aligned with flow cytometry showed recruitment and activation of LysM^+^ cells in the dorsal pachymeninx at 5 and 10 hours following intranasal infection. Imaging of the cribriform plate suggested that both pneumococci and microspheres entered through the foramina via an inward flow of fluid connecting the nose to the pachymeninx. Our findings bring new insight into the varied mechanisms of pneumococcal invasion of the central nervous system, but they are also pertinent to the delivery of drugs to the brain and the entry of airborne particulate matter into the cranium.

## INTRODUCTION

There is growing literature providing evidence that the brain and central nervous system (CNS) could be direct tissue targets upon inhalation of microorganisms or particulate matter, resulting in neurological and other inflammatory disorders (1–4). A wide array of microorganisms are shown to be present in the meningeal or parenchymal compartments (4). Streptococcus pneumoniae (pneumococcus) is a frequent asymptomatic coloniser of the human nasopharynx (5); it can spread from there to invade other tissues including the lungs, blood, and the cranium (1, 6, 7). However, the mechanisms by which neurotropic microorganisms, such as the pneumococcus, gain access into the CNS remain obscure.

**FIG 1:**
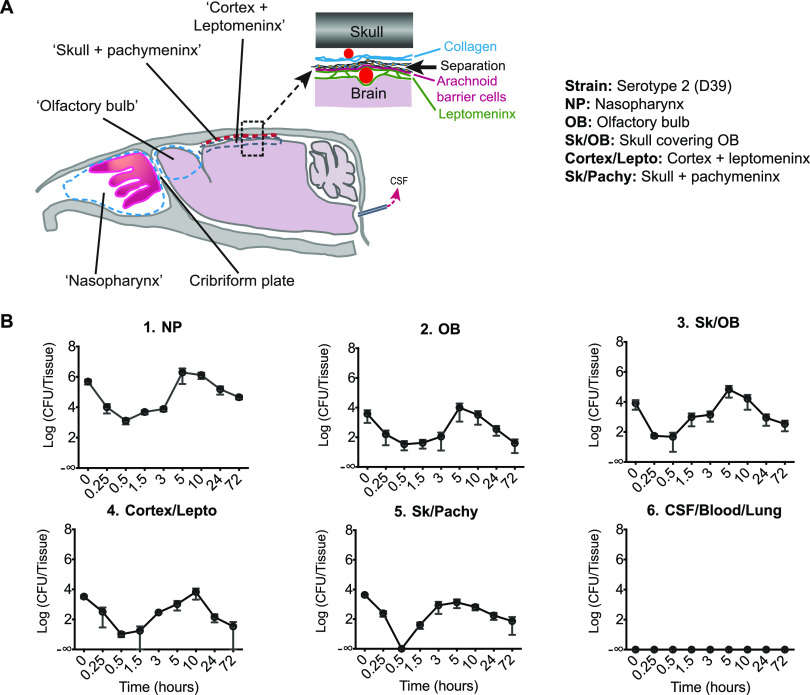
Pneumococci intranasally instilled reach the meninges, by-passing the blood systemic circulation. (A) Schematic representation showing the situation of the tissues investigated. CSF, cerebrospinal fluid. Top right inset: Schematic magnification of the meninges of the dorsal brain showing the outer layer, the pachymeninx, which contains the collagenous dura mater, the inner layer, the leptomeninx, containing the subarachnoid space, and the underlying brain cortex. Both layers contain blood vessels (red circles). (B1 to B6) Pneumococci (Serotype 2, strain D39) were intranasally administered and CFU counted between 0 and 72 hours in tissue samples including the nasopharynx (NP), the olfactory bulb (OB), the skull and its adhering OB tissue (Sk/OB), the cortex and its adhering leptomeninx (lepto), the skull and its adhering pachymeninx (SK/pachy), the CSF, whole blood, and lungs. Data are shown as mean ± SEM (*n* = 5 mice per time point).

Intracranial invasion in humans is not routinely examined until clinical symptoms have developed, so there is no direct evidence of the route of initial invasion. In mice, where tissues can be examined at shorter or predefined time points, it has been reported that direct instillation of pneumococci into the nasal cavity can lead to invasion of cranial tissues in the absence of bacteremia. Marra and Brigham (8) examined homogenized brains of infant rats 1 hour after nasal instillation and found colony forming units (CFU). Rake (9), van Ginkel et al. (10), and Hatcher et al. (11) found bacteria in brain tissue also in the absence of blood infection, with higher bacterial density in the frontal, olfactory area. This supports the hypothesis that nonhematogenous translocation of bacteria from the nasal cavity to olfactory tissue is through the foramina of the cribriform plate of the ethmoid bone, which allow passage of the olfactory nerve bundles (12, 13). It has been suggested that bacterial invasion and inflammation are in the inner layers of the meninges, i.e., the leptomeninx, which is composed of the pia, the subarachnoid space, and the arachnoid (6, 14, 15) (Fig. 1A). When pneumococci are found in the cerebrospinal fluid (CSF), this suggests its presence in the subarachnoid space of the leptomeninx. However, fluid from the outer meningeal layer, the pachymeninx, which contains the collagenous layers that constitute the dura mater (16, 17), is not normally sampled in the clinic.

The pachymeninx is richly innervated and vascularized, and contains lymph vessels, and, at least over the cortical convexities, is thicker than the leptomeninx (18–22). Inoculation of pneumococci into the “subdural space” of the pachymeninx is an effective route of administration (16, 23, 24). The subdural space is now thought to be a virtual space within the pachymeninx situated beneath layers of collagen and above the dural border cells that overlie the arachnoid barrier layer (16, 20, 21, 25, 26). Here, we have distinguished the meninges from the brain tissue and report that, at least at early times postintranasal infection, pneumococci reach the dorsal meninges in the pachymeningeal compartment rather than the leptomeninx, inducing an immune response there. To see if translocation from the nasopharynx to meninges depended on an active biological feature of pneumococci, we also looked for (and found) translocation of inert polystyrene microspheres and compared a range of diameters. As well as for microbes, inward translocation from nose to brain is known to occur for stem cells (27) and for nonbiological particles including neurotherapeutics (28, 29) and airborne particulate pollutants (30). Our results outline a novel pathway of entry into the central nervous system that may be common to all of these materials.

## RESULTS

### Nasopharyngeal translocation of pneumococci following intranasal infection.

In order to characterize nasopharynx-to-meninges translocation over time, we intranasally infected mice and collected tissue over a period of up to 72 hours postinfection (Fig. 1B1 to B6). All the mice showed localized invasion, in nasopharynx and surrounding tissues, but at no time point was bacteremia detected, nor were CFU found in CSF samples recovered from the cisterna magna or in lung tissue (Fig. 1B6). At the earliest time point, when mice were culled 2 minutes after intranasal challenge and the tissue dissected immediately, CFU were found not only in the nasopharynx (Fig. 1B1) but also in the olfactory bulb and attached meninges (Fig. 1B2). Since the translocation from nasopharynx to cranium was so fast (minutes), it is unlikely that it involved damage to cells of the nasopharynx. Apart from the fact that there was no detectable release of pneumococci to the blood, a number of studies have indeed shown that pneumococci cause detectable damage to cells only after several hours of exposure (31–39). Surprisingly, CFU were also recovered at this earliest time point from the more remote tissue associated with the dorsal skull and cortex. In all these tissues, the numbers of CFU then fell by about two log units to reach a minimum at the 30-minute time point, even, in the skull/pachymeninx sample, becoming undetectable. The numbers of CFU then increased with a doubling time of less than 20 minutes to reach a peak at 5 to 10 hours, before falling again. CFU counts then declined gradually over time, but persisted up to 14 days postinfection, including in the brain and leptomeninx for both D39- and serotype 1 (ST217)-challenged mice (Fig. S1).

10.1128/mbio.01024-22.1 FIG S1 Long-term monitoring of pneumococci after intranasal administration. (A) Pneumococci serotype 1 (S1, sequence type 217) (A) or serotype-2, strain D39 (NCTC 7466) (B) were intranasally administered in 10 μl inocula containing approximately 10^8^ CFU. CFU were determined at 15 min, 1 day, and subsequently to 14 days postinfection in various tissues. Data are shown as mean ± SEM (*n* = 5 mice per time point). Between day 0 and 14, an approximate 3-log decline was observed in the CFU counts in all of the collected tissues. S1-infected mice started showing clearance at day 14 in the olfactory bulb (2/5 mice) and in the brain/leptomeninx (3/5 mice). No viable pneumococci were detected in the blood or lung. Note that because no measurements were made between 15 minutes and 24 hours, any bottleneck at about 1 h, as in Fig. 1B, would have been missed. Download FIG S1, EPS file, 1.1 MB. Copyright © 2022 Audshasai et al. 2022 Audshasai et al. https://creativecommons.org/licenses/by/4.0/ This content is distributed under the terms of the Creative Commons Attribution 4.0 International license.

### Pneumococci are predominantly found in the outer meninges following translocation from the nasopharynx.

**FIG 2:**
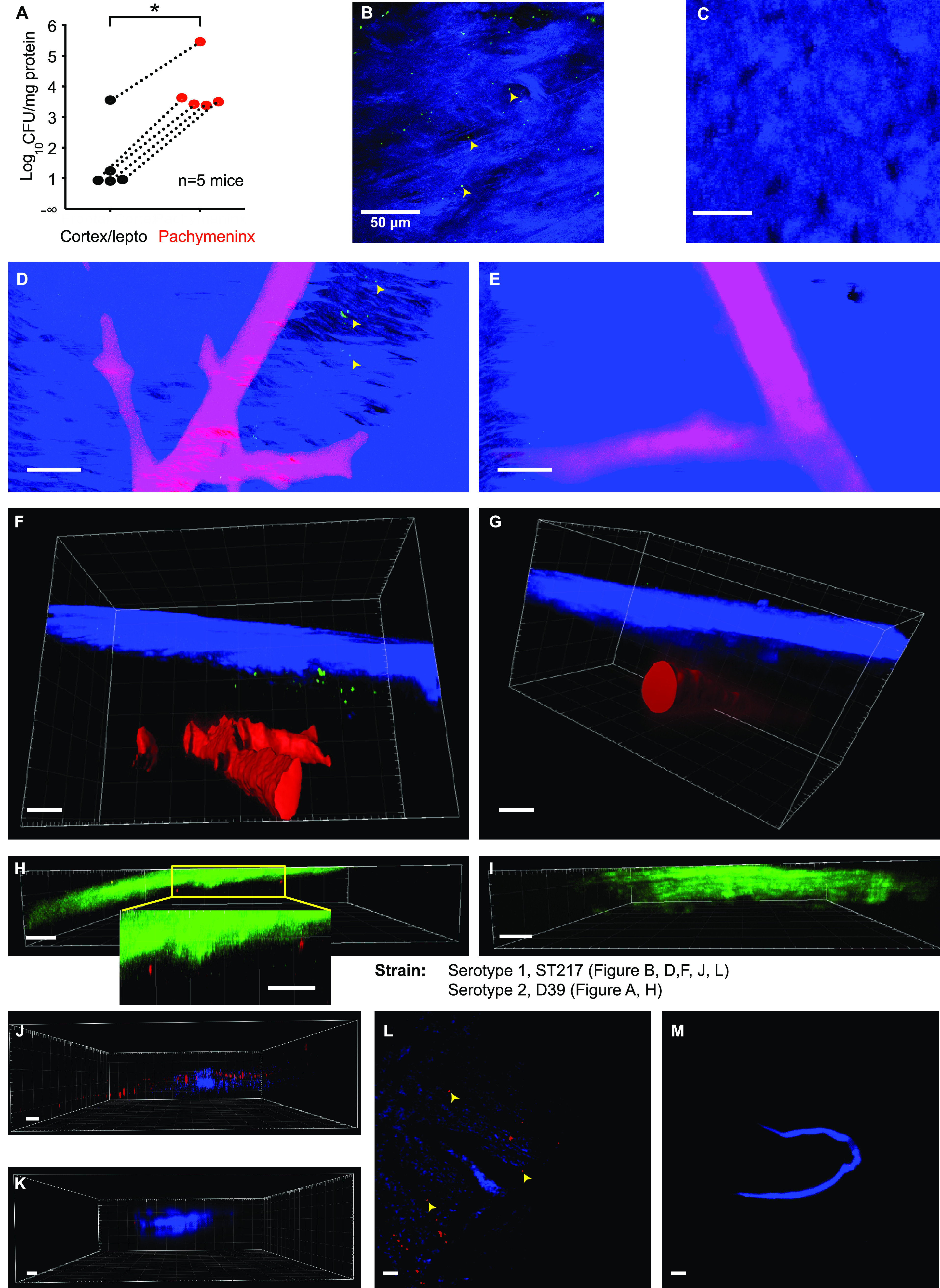
Intranasally administered pneumococci are rapidly and predominantly found in the pachymeningeal compartment of the dorsal meninges. (A) Pneumococci D39 were applied to the nose and the mice euthanized 10 hours later. The skull and brain were separated, and CFU were counted for tissue from the superficial cortex + attached tissue and for tissue scraped from the skull (“pachymeninx”). The numbers of CFU were counted and expressed relative to the weight of protein per tissue sample. In the infected mice, the density of CFU was much higher in the tissue scraped from the skull. (B) CFSE-labeled serotype 1 (ST217) pneumococci at 90 minutes postinfection in tissue adhering to the skull after removal of the brain and imaged from the intracranial face (the “amaguri” preparation of Toriumi et al. [40]). Excitation was by laser at 840 nm, which produced two-photon excitation of CFSE (green), and second harmonic generation (SHG) (blue) from collagen and skull bone. Image is representative of *n* = 3 mice. Maximum intensity z-projection, z = 171 μm. (C) Under identical imaging conditions to panel B, no green particles were detected in an uninfected naive mouse. Maximum intensity z-projection, z = 178 μm. (D, F) CFSE-labeled serotype 1 (ST217) S. pneumoniae were instilled in the nose of a mouse. Thirty minutes later, the mouse was killed with CO_2_ and perfused with DiI to label blood vessels (Li et al. [42]). Imaging was done through the skull and into the meninges. CFSE-labeled Sp (green) are seen in a z-projection 444 μm deep (D). In a 3D projection, Sp are seen close to the skull (blue: SHG) and above large blood vessels (red), but are absent from deeper layers (F). (E and G) Under identical imaging conditions to (F), no green particles were detected in an uninfected mouse. Z-stack (E) and 3D representation (G). Scale bar = 50 μm. (H) S. pneumoniae D39 expressing mKate were instilled in the nose. After 3.7 hours, the dorsal meninges and underlying brain were imaged *in vivo* through the skull with excitation at 1,140 nm. The SHG from skull bone and collagen is green and emission from mKate is red. A representative xz-section including two groups of pneumococci is shown. (I) Similar red signals were not seen in uninfected mice under the same imaging conditions as in panel H. (J and K) Serotype 1 (ST217) S. pneumoniae stained with BacLight Red were instilled in the nose. At 15 minutes postadministration, mice were perfused transcardially with PBS followed by fixing solution (4% PFA). Dorsal skull mounts were stained with anti-LYVE1 antibody and imaged on the skull bone-oriented surface with excitation at 561 and 405 nm. A representative xz-projection of the skull whole mount is shown for the Sp-infected (J) and uninfected (K) mouse. (L and M) Maximum intensity z-projection of the images shown in (J) and (K), respectively, z = 16.75 μm and z = 340.37 μm. *, *P* < 0.05.

To quantify the density of viable pneumococci in the outer (pachy-)meningeal tissue, the skull was separated from the brain and the tissue attached to its inner surface was scraped off. The pneumococcal density was expressed as the number of CFU per milligram protein content. This was compared with the density in the superficial cortex and attached leptomeninx. Eight mice were inoculated intranasally. Ten hours later, pachymeningeal tissue and tissue including leptomeninx and cortex were analyzed for CFU. CFU were found in samples from five mice: the mean number in the pachymeningeal samples was at least 2 log units higher than in the cortex/leptomeningeal samples (*P* = 0.019) (Fig. 2A). This shows that pneumococci were far more concentrated in the pachymeninx than in the superficial cortex and attached meninges. Indeed, if the separation occurred in the inner layers of the pachymeninx, it is possible that all the intracranial CFU in these mice were in the pachymeninx, the CFU in the “cortex” samples coming from contaminating remnants of the inner pachymeninx.

To obtain further information on the location of intracranial pneumococci, we used two-photon microscopy and fluorescent bacteria. In the first method, pneumococci were labeled in culture by uptake of carboxyfluorescein succinimidyl ester (CFSE) (Fig. S2A) and then delivered intranasally to mice. Thirty minutes later, mice were culled, the brain was removed, and a piece of dorsal skull bone with adherent tissue was imaged from the intracranial side (40). With femtosecond excitation at 840 nm, sparse particles emitting green fluorescence were visible (Fig. 2B); these were in the same plane as the collagen fibers of the dura made visible by second harmonic generation (SHG) (41). Green particles were not seen in uninfected (i.e., naive) mice (Fig. 2C). To see if the distribution of pneumococci extended deeper under the skull than the pachymeninx, CFSE-labeled pneumococci were also imaged through the skull into the intact meninges and superficial parenchyma. To provide anatomical markers, blood vessels were labeled by intravenous infusion of the carbocyanine dye DiI (42). Again, fluorescent particles were observed in tissue from infected mice (Fig. 2D) but not uninfected ones (Fig. 2E). In three-dimensional (3D) reconstructions, it was evident that the green particles in infected mice were close to the skull and above the pial blood vessels (Fig. 2F and G). Although endogenous fluorescent particles can often be seen with two-photon microscopy, their detection requires a higher excitation intensity and detector sensitivity than those used here. Nevertheless, to check that the CSFE-labeled pneumococci were not confused with endogenous fluorescent objects, we also used pneumoccci with very different excitation and emission spectra. Mice were infected with pneumococci expressing the red fluorescent protein mKate2 (43). Ten hours later, the dorsal meninges and superficial cortex were imaged *in vivo* through the skull using two-photon excitation at 1,140 nm. Red particles were observed close below the green SHG of the skull of infected mice (Fig. 2H) but not in uninfected mice (Fig. 2 I).

10.1128/mbio.01024-22.2 FIG S2 Fluorescence microscopy images of Streptococcus pneumoniae. (A) Two-photon fluorescence image of S. pneumoniae serotype 1, sequence type 217 loaded with CFSE for 45 min. The fluorescent Sp are bright and clearly distinguishable in either monococci (yellow arrows) or diplococci (red arrows). (B) Confocal fluorescence image of S. pneumoniae sequence type 217 loaded with red-fluorescent BacLight Red for 15 min. The fluorescent Sp are bright and clearly distinguishable in either monococci (yellow arrows) or diplococci (green arrows). Download FIG S2, EPS file, 1.8 MB. Copyright © 2022 Audshasai et al. 2022 Audshasai et al. https://creativecommons.org/licenses/by/4.0/ This content is distributed under the terms of the Creative Commons Attribution 4.0 International license.

To examine the location of pneumococci in relation to dural lymph vessels, we used another label to stain pneumococci (BacLight Red; Thermo Fisher [44, 45]), which appear as dots in the meninges of infected mice (Fig. 2J to K; Fig. S2B), while none were found in uninfected mice (Fig. 2L and M). Overall, our results clearly show that pneumococci that reach the pachymeninx following translocation from the nasopharynx, are located outside, not inside, LYVE-1^+^ structures. Since the meningeal lymph vessels are in the pachymeninx (46–48), the presence of a LYVE-1 signal within tens of micrometers of the BacLight Red-356-labeled pneumococciis further evidence that pneumococci are in the pachymeninx.

**FIG 3:**
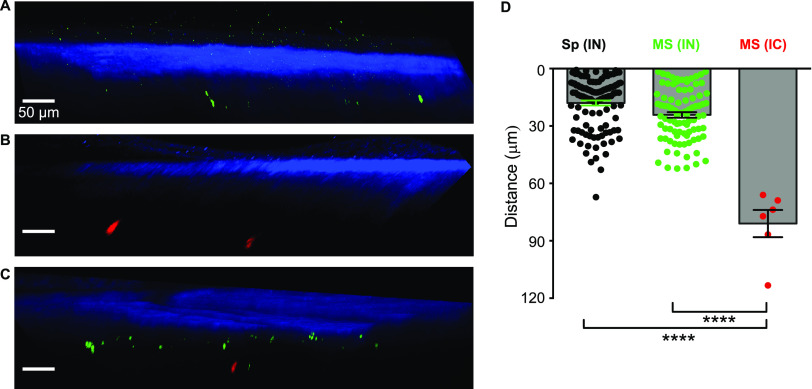
Microspheres administered intranasally were found in a layer close to the skull. (A to C) One-micrometer-diameter green fluorescent microspheres were applied to the nose of a mouse (A) and 2-μm Nile Red-labeled microspheres were injected in the cisterna magna of another mouse (B). (C) A third mouse was subjected to both procedures, i.e., intranasal administration of 1-μm green fluorescent microspheres followed immediately by intracisternal injection of 2-μm Nile Red-labeled microspheres. In each case, 30 minutes after the infection, the mouse was euthanized, and the meninges and cortex were examined through the skull with two-photon microscopy. (A to C) Microspheres administered intranasally were only found in a layer close to the skull (A and C) while those infused in the cisterna magna were deeper (B and C). Scale bar = 50 μm. (D) The distances from the skull of all fluorescent Sp (black circles) measured in the 3D reconstructions measured postmortem as for Fig. 2, panels D to I. The distances from the skull of Sp and fluorescent microspheres measured in the 3D reconstructions measured postmortem as for Fig. 3, panels A to C, upon intranasal instillation of Sp (black circles, *n* = 120 signals, imaging of 4 mice), or upon intranasal (green circles, *n* = 90 signals, imaging of 4 mice) or intracisternal (red circles, *n* = 6 signals, imaging of 3 mice) administration of microspheres. ****, *P* < 0.0001. IN, intranasal; IC, intracisternal; MS, microspheres; Sp, Streptococcus pneumoniae.

We next sought to determine the distance from the skull of the fluorescent signals. The mean measured distance of pneumoccci (CFSE and mKate2 labeled) was 18.2 μm, SD 13.6 μm, and *n* = 120 particles measured in four z-stacks from four mice (Fig. 3D, Sp black circles). Although the large blood vessels in the pachymeninx and in the leptomeninx make the thicknesses of both layers very variable (20), the distribution of depths suggests that most pneumococci were in the pachymeninx, and certainly not within the brain parenchyma.

We found that robustly fluorescent polystyrene microspheres of diameter 1 μm were also transported from the nasopharynx to the meninges so we used these as a surrogate for pneumococci. It is known that pneumococci and molecules infused in the mouse cisterna magna are carried by CSF to spaces in the leptomeninx (20, 49, 50). Hence, to make another test of whether particles entering from the nasopharynx were arriving in this space or in the pachymeninx, we applied green fluorescent microspheres to the nose and infused red fluorescent microspheres into the cisterna magna of the same mouse. Thirty minutes later, the mouse was euthanized and the meninges and cortex were examined through the skull with two-photon microscopy (Fig. 3A to C). The microspheres administered intranasally were found at a mean distance from the skull of 24.2 μm, SD 13.6 μm, and *n* = 90 particles measured in four z-stacks in four mice, which was not significantly different from that of the bacteria (Fig. 3D). The mean depth of those infused in the cisterna magna was 81.0 μm, SD 15.9 μm, and *n* = 6 particles measured in three z-stacks in three mice, which is more than three times greater (Fig. 3D). Altogether, the various techniques we used concur in showing rapid pneumococcal translocation from the nasopharynx to the pachymeninx (rather than the leptomeninx) of the dorsal meninges.

### Pneumococcal infection causes recruitment of innate immune cells to the dorsal pachymeninx.

**FIG 4:**
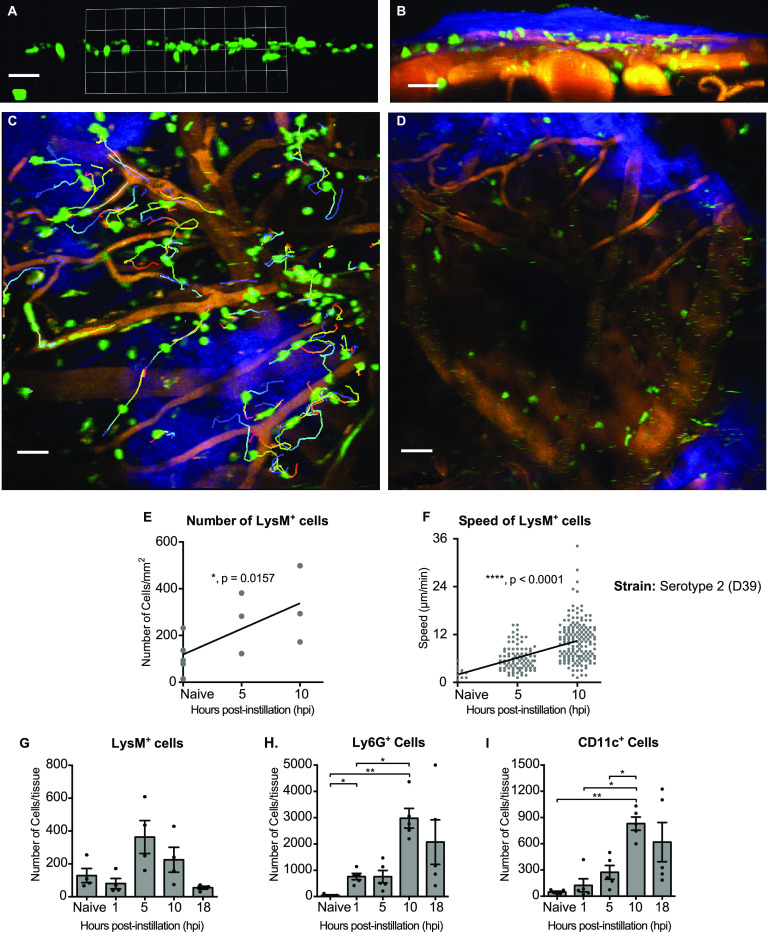
Intranasal instillation by S. pneumoniae leads to transient recruitment and activation of LysM^+^ in the calvarial pachymeninx. (A and B). *In vivo* two-photon imaging shows that nearly all intracranial LysM^+^ cells are in the meninges. (A) Horizontal view of a 3D reconstruction from a z-stack of an uninfected mouse showing only LysM^+^GFP cells, which lie in a shallow layer. (B) A different view of the 3D image in panel A showing, in addition to the LysM^+^GFP cells (in green), the skull bone (in blue: SHG), nuclei of the pachymeninx (blue from intravenous injection of furamidine), and blood vessels (shown orange-yellow, labeled with rhodamine). Excitation at 840 nm. (C) Tracks of LysM^+^ cells in the meninges of a mouse imaged at 10 hours after intranasal administration of pneumococci serotype 2 D39; z-projection of z-stacks at 23 μm deep; time series for 15 min. (D) Tracks of LysM^+^ cells in the meninges of an uninfected mouse under the same imaging conditions as panel C: z-projection at 30 μm deep; time series for 32 min. All images shown in panels A to D were acquired at 10 hour postchallenge. (E) Numbers of LysM^+^ GFP cells per unit area of the meninges counted in *in vivo* images. Each point was obtained from one z-stack. The linear regression line has a slope greater than one with *P* = 0.016. (F) Mean track speeds of mobile GFP^+^ cells in the same imaging conditions as in panel E. (G to I) Flow cytometry of cells from tissue scraped from the calvarial skull. Cells selected as CD45^+^, CD4^+^, and CD11b^+^ were further sorted into LysM^+^ (G), Ly6G^+^ (H), or CD11c^+^ (I) cells. Each dot represents one mouse; error bars are SEMs; *, *P* < 0.05; **, *P* < 0.01.

In extracranial tissues such as lung and spleen, pneumococci and neutrophils interact vigorously (51–54). To see how neutrophils reacted to arrival of pneumococci in the dorsal meninges, we imaged them *in vivo* by intravital two-photon microscopy through the thinned skull of mice expressing enhanced green fluorescent protein (eGFP) under the control of the LysM promoter (Fig. 4A to D). In addition to neutrophils, LysM is expressed in other cells of the myelomonocytic lineage, but in *LysM^GFP/GFP^* mice, neutrophils are the brightest (55) and can be easily distinguished from macrophages (52, 56). In agreement with others, we found very few LysM^+^ cells in uninfected mice (56–59) (Fig. 4D and E), confirming that the skull-thinning and two-photon imaging did not recruit myelomonocytic cells to the meninges within the duration of the experiment (58). After intranasal challenge with pneumococci, the number of LysM^+^ cells in the dorsal meninges was increased at 5 and 10 hours (Fig. 4E). Nearly all of them were in a layer close under the skull, in the plane of smaller vessels typical of the pachymeninx (60, 61) (Fig. 4C and D), and above the pia and parenchyma (Fig. 4A and B) where the microglia are present (62). Analysis of videos (Supplemental Movies S1 and S2) showed that motile LysM^+^ cells moved at progressively higher mean speeds as time progressed (Fig. 4F) (see also z-projections of tracks in Fig. 4C and D). The mean speed of motile LysM^+^ cells at 10 hours postinstillation was 10.4 ± 0.4 μm/min, which is close to the 9.7 μm/min found by Kreisel et al. (52) for neutrophils in mouse lung. Many LysM^+^ cells in the pachymeninx followed generally directed trajectories, some along the outsides of blood vessels (Fig. 4C and Supplemental Movie S2) rather than making random walks (52). The averaged x-, y-, and z-components of the velocities were not significantly different from zero, i.e., no global drift was detected.

10.1128/mbio.01024-22.5 MOVIE S1 Intravital imaging of an uninfected LysM-eGFP mouse. Two-photon imaging through thinned skull, under the same experimental conditions as Fig. 4D. Download Movie S1, MPG file, 0.7 MB. Copyright © 2022 Audshasai et al. 2022 Audshasai et al. https://creativecommons.org/licenses/by/4.0/ This content is distributed under the terms of the Creative Commons Attribution 4.0 International license.

10.1128/mbio.01024-22.6 MOVIE S2 Intravital imaging of an Sp-infected LysM-eGFP mouse. Two-photon imaging through thinned skull at 10 hours postinfection, under the same experimental conditions as Fig. 4C. Download Movie S2, MPG file, 1.0 MB. Copyright © 2022 Audshasai et al. 2022 Audshasai et al. https://creativecommons.org/licenses/by/4.0/ This content is distributed under the terms of the Creative Commons Attribution 4.0 International license.

To determine the changes in numbers of LysM^+^ cells in the dorsal pachymeninx over a wider range of times (0 to 18 hours) we used flow cytometry of tissue scraped from the dorsal skull of C57BL/6 wild-type nonreporter mice. In addition to LysM (Fig. 4G), other gating was used to select cells expressing Ly6G, an integrin-binding protein strongly expressed only on neutrophils (63, 64), and also CD11c, a marker of dendritic cells (DCs) and macrophages (65, 66) (Fig. 4H and I). In agreement with the intravital imaging (Fig. 4E), the number of LysM^+^ increased over time (5 to 10 h) then tended to decrease (Fig. 4G and H). The time course of Ly6G expression appears to be delayed compared to that of LysM. Since the expression of the *lysM* gene is driven differently from that of *ly6G*, changes in their relative quantities might be expected as the neutrophil population responds to the presence of pneumococci (55, 67). As well as LysM^+^ cells, the number of CD11c^+^ cells also increased after intranasal challenge (Fig. 4I). In videos of the dorsal meninges of infected CD11c-eYFP reporter mice (Supplemental Movie S3), nearly all the yellow fluorescent protein-positive (YFP^+^) cells displayed a rapid extension and retraction of dendrites, suggesting that they, and therefore most of the CD11c^+^ cells of Fig. 4I, were dendritic cells. The number of CD11c^+^ cells in the dorsal pachymeninx increased some 10-fold, to a peak at about 10 hours (Fig. 4I). This increase is much earlier than that reported in the nasopharynx and nasal-associated lymph nodes, which was insignificant until 3 weeks after nasal infection with pneumococci (68). It is also much faster than the increase in pachymeningeal DCs caused by trypanosomiasis, which occurs between 5 and 10 days after infection (41).

10.1128/mbio.01024-22.7 MOVIE S3 Intravital imaging of a Sp-infected CD11c-eYFP mouse. Two-photon imaging through thinned skull at 3.5 hours postinstillation, excitation at 960 nm with ×20, N.A. 1.0 water immersion objective. Detector channels were set at <490 and 570 nm for second harmonic generation and CD11c, respectively. Z-stacks = 24 μm deep and total acquisition duration = 27 min. Download Movie S3, MPG file, 8.1 MB. Copyright © 2022 Audshasai et al. 2022 Audshasai et al. https://creativecommons.org/licenses/by/4.0/ This content is distributed under the terms of the Creative Commons Attribution 4.0 International license.

### The speed of translocation from nasopharynx to meninges is size-dependent.

**FIG 5:**
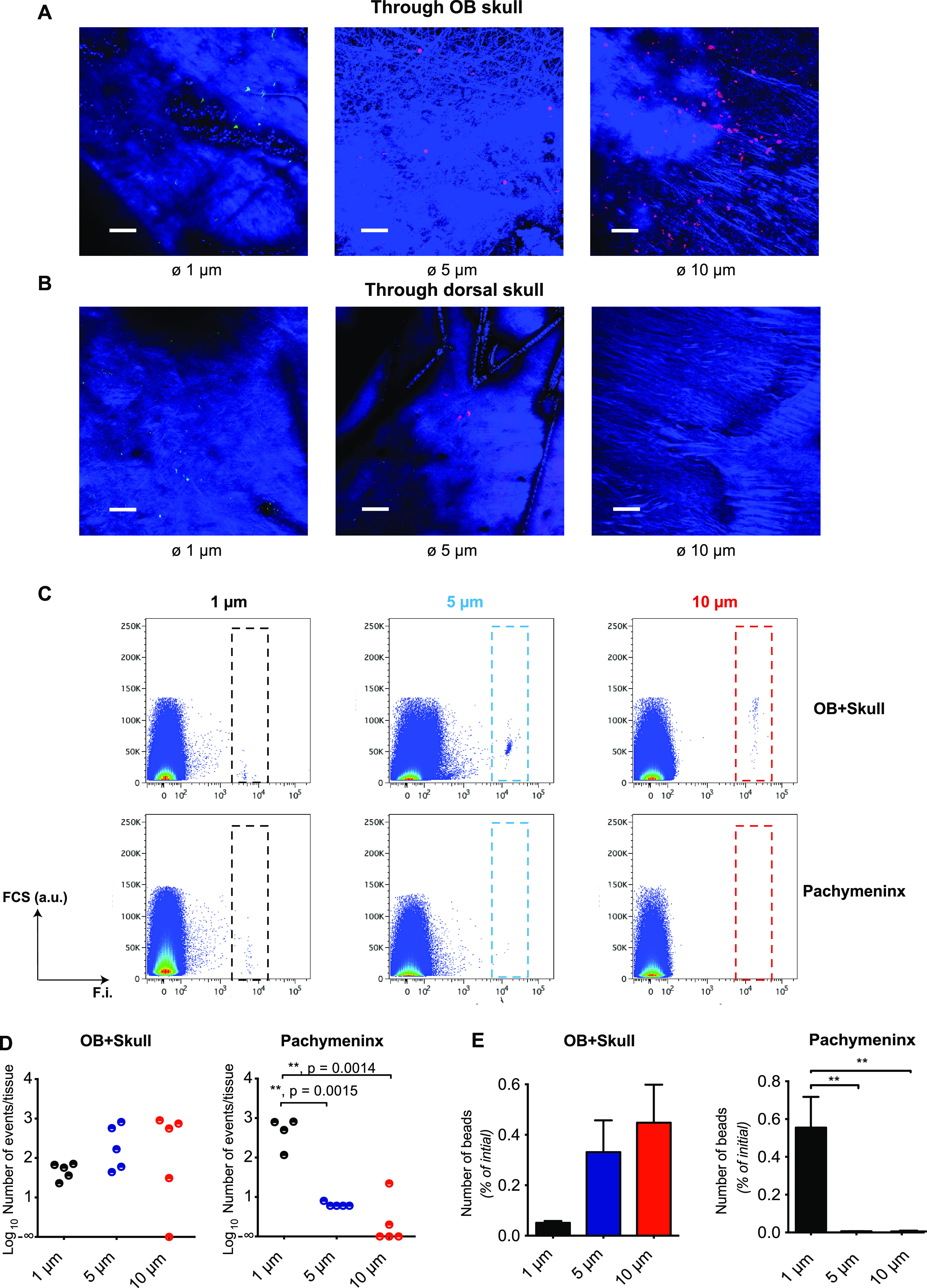
The speed of transit from nose to calvarial meninges is lower for larger microspheres. Transport from the nose of fluorescent microspheres of three diameters was examined: 1 μm (yellow-green), 5 μm, and 10 μm (Nile Red); 10^5^ microspheres in 10 μL were applied to the nose and the mice were killed 30 minutes later. (A and B) Two-photon imaging *ex vivo* through the skull and into the meninges in the areas of the olfactory bulb (A) and the dorsal brain (B). Z-stacks were 154 μm deep. Excitation was at 840 nm, which produced fluorescence from the microspheres and blue SHG from bone and collagen. (C to E) For each of the three diameters of microsphere (1, 5, and 10 μm), five mice were inoculated. 30 minutes later, pachymeningeal tissue was scraped from the skull covering the olfactory bulb and from the dorsal skull, and the numbers of microspheres counted by flow cytometry. F.I., fluorescence intensity; a.u., arbitrary units. The numbers of beads detected by flow cytometry for each tissue sample (within the dashed rectangles in panel C) are plotted in panel D where each dot represents one mouse. To better illustrate the dynamics of the translocation, the numbers were then expressed as percentages of the number (10^5^) of microspheres applied to the nose and plotted on a linear scale (E). Error bars indicate SEMs, one-way ANOVA followed by Tukey’s *post hoc* test, **, *P* < 0.01.

Unlike pneumococci, chemically inert microspheres are not susceptible to destruction by the host defense cells or by fixation of the tissue, nor can they multiply; hence, tracking is simplified. In addition, the brightness and stability of the microsphere fluorescence give more confidence that the signals detected by microscopy were not artifacts. Figure 3A and C shows that fluorescent polystyrene microspheres with a diameter of 1 μm, similar to that of pneumococci, reached the dorsal pachymeninx from the nasal cavity in under 30 minutes. Since they reached the same destination as pneumococci and with similar rapidity, we hypothesized that they may have been transported in the same way. To obtain clues to the mechanism of translocation, we asked if it could support microspheres of diameter greater than 1 μm. We therefore tested and compared the translocation of microspheres of diameters 1, 5, and 10 μm. At 30 minutes after nasal administration, microscopic observation of microspheres in the meninges overlying the olfactory bulb suggested abundance in the order 1 μm < 5 μm < 10 μm (Fig. 5A) while, in contrast, in the dorsal meninges the order of abundance was 1 μm > 5 μm > 10 μm (Fig. 5B). These distributions were quantified by flow cytometry on pachymeningeal tissue scraped from the two areas of the skull (Fig. 5C). In contrast to the CFU (Fig. 1B), at 30 minutes the number of 1-μm microspheres in the dorsal pachymeninx was higher than in the olfactory bulb + skull tissue. The number of 1-μm microspheres present in the dorsal pachymeninx was 0.55 ± 0.16% of the number instilled in the nares. This is some 100-fold higher than that of microspheres with diameters of 5 μm (0.0064 ± 0.0004%) and 10 μm (0.0054 ± 0.004%) (Fig. 5D and E). Conversely, in the pachymeningeal tissue above the olfactory bulb, no significant differences were found between the three sizes of microspheres (Fig. 5D and E). The abundance of 5- and 10-μm microspheres in the pachymeninx of the olfactory bulb, compared to their paucity in the dorsal pachymeninx, with the opposite being true for 1-μm microspheres, shows that the transport of the larger microspheres is hindered. Since the data are limited to one time point, it is not possible to say if the hindrance of the larger microspheres is uniform along the pathway (i.e., they travel more slowly) or if the size-dependent hindrance occurs at one anatomical stage of the translocation.

**FIG 6:**
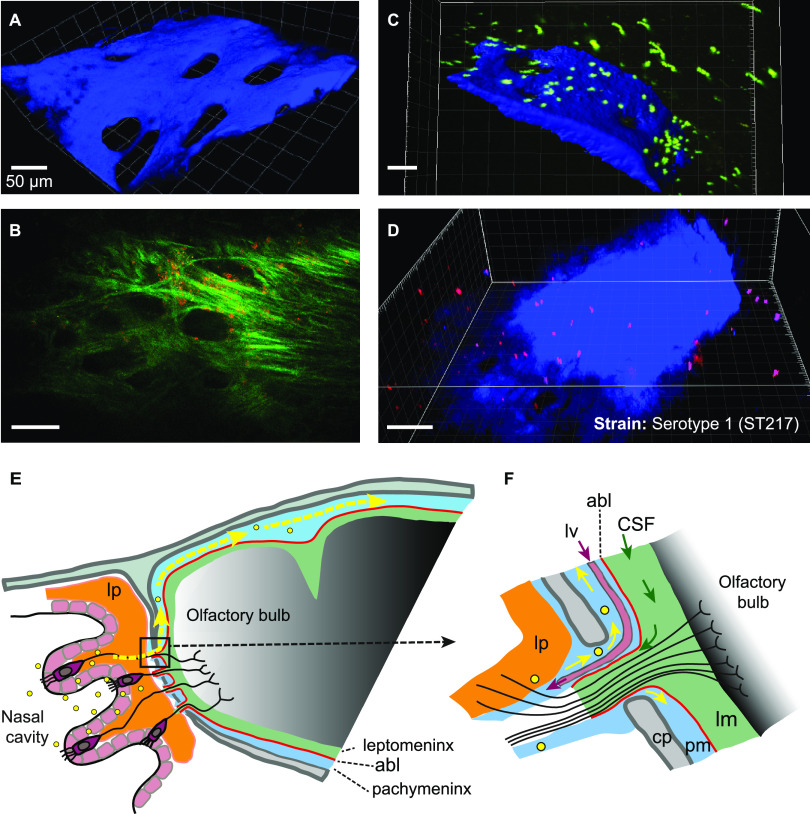
Microspheres and S. pneumoniae on the cribriform plate. Two-photon images of the intracranial face of freshly dissected ethmoid bone covered by a layer of olfactory bulb tissue (which gives no signal). (A) Excitation at 900 nm gives blue SHG from the cribriform plate. This was a naive LysM-GFP reporter mouse. (B) Excitation at 1140 nm gives an image of collagen-like fiber structures, presumably dura mater. This was a CD11c-YFP reporter mouse. YFP is poorly excited at 1140 nm but faint CD11c^+^ cells can be seen. (C and D) 3.8 × 10^7^ yellow-green fluorescent polystyrene microspheres of 1 μm diameter size (green) (C) or BacLight Red-stained serotype 1 (ST217) pneumococci (red) (D) were administered intranasally. The mice were culled at 30 minutes, and the intracranial face of the cribriform plate (SHG: blue) was imaged with excitation at 840 nm. Baclight Red was detected at 571 to 664 nm. Microspheres and pneumococci are seen close to the bone; some microspheres are drifting in the superfusate. (E) Scheme (not to scale) of the anatomy of the pathway (partly hypothetical, sagittal section). Olfactory neurons with their cell bodies in the olfactory epithelium send axons through the foramina of the cribriform plate where they are surrounded by cells which have been described as “olfactory ensheathing cells” (12, 134–137) or as forming extensions to some, or all, of the pia, the arachnoid, the dura, and the periosteum (27, 138–140). Microspheres and pneumococci (yellow dots) are transported through the cribriform plate and are found in the pachymeninx (yellow arrows), which is separated from the leptomeninx by the arachnoid barrier layer (abl; red line). (F) Enlargement of the dashed rectangle in panel E. CSF flows out of the subarachnoid space of the leptomeninx (lm) along extracellular spaces in a bundle of olfactory nerve fibers that traverses a foramen of the cribriform plate (cp). Lymph draining from the pachymeninx flows out (46, 119) through a lymph vessel (lv), Sp, and microspheres are carried into the pachymeninx (pm) along a space adjacent to the lamina propria (lp) (Galeano et al. [27]). The arachnoid barrier layer (abl) is indicated by a red line.

To see if pneumococci and microspheres were passing through the cribriform plate, we removed brain tissue from above the ethmoid bone postmortem until the remaining layer was thin enough to allow two-photon imaging through its depth and into the cribriform plate and its overlying tissue. The foramina were clearly visible (Fig. 6A), and the overlying collagen of the dura mater appeared also to have holes that could allow passage of olfactory nerve bundles (Fig. 6B). In other mice, we administered fluorescent pneumococci or microspheres to the nose and culled the mouse at 15 minutes. Imaging of the cribriform plate and overlying tissue showed pneumococci and microspheres very close to the bone (Fig. 6C and D); they moved slowly, if at all. This supports the conclusion that pneumococci and microspheres passed through the cribriform plate and entered the pachymeninx. Some microspheres were in the superfusate and drifted with its thermal convection (squiggles in Fig. 6C). This shows that they were not trapped inside cells.

## DISCUSSION

Intracranial invasion by Sp is often, although not always, associated with the presence of pneumococci in the patient’s blood (69, 70) and/or attached to the cells forming the blood-brain barrier (71). Observations such as these have led to the widespread opinion that invasion of the central nervous system by pneumococci occurs following bacteremia (4, 6, 69, 71–77). Here we have shown that, in mice, pneumococci can translocate across the nasopharyngeal tissue to the meninges without detectable bacteremia. We have made two key novel findings: that translocation of pneumococci into the outer meninges of the pachymeninx occurs very rapidly (within 2 min) and that size is the key factor in this process.

Previous studies did not examine the dorsal meninges earlier than 1 hour after intranasal administration of material of any kind. At 1 hour, Clark (78) found Prussian blue in the pachymeninx, and Galeano et al. (27) found stem cells at 2 hours, as we found for pneumococci and microspheres (Fig. 1B, 2, and 5). The prime evidence that pneumococci were in the pachymeninx, rather than the leptomeninx, is the proximity of pneumococci to the skull (Fig. 2A to I) and to LYVE-1^+^ structures (46, 47) (Fig. 2J to M). This finding is supported by the absence of viable pneumococci in the CSF at 72 hours postinfection, which suggests that pneumococci did not breach the arachnoid barrier layer and reach the CSF channels in the leptomeninx (20, 25, 61, 79) (Fig. 6E and F).

It is important to highlight that the absence of viable pneumococci in the CSF may raise questions on the pathological importance of our finding. Indeed in the clinic, acute bacterial meningitis is not normally diagnosed if viable bacteria are absent from the CSF (80, 81). However, a number of reports suggest that CSF-negative cultures do not rule out an intracranial bacterial pathology (82–84), nor does the absence of clinical symptoms (85–88). In our study, the flow cytometry analysis of Ly6G^+^ cells and the intravital imaging of LysM^+^ cells showed that the number and mean speed of LysM^+^ cells in the dorsal pachymeninx increase up to 10 hours postinfection, these events being hallmarks of local inflammation and immune cell activation. Although the recruitment of LysM^+^ cells appears to be too slow to account for the rapid fall in CFU over the first 30 minutes in the dorsal skull preparation, after the subsequent rebound, the number of CFU begins to fall again at less than 5 hours post instillation, as the LysM^+^ population approaches its maximum. It appears, therefore, that the LysM^+^ population increases until the clearance of pneumococci is established (54, 89) and then begins to fall. Intense immune reactions in the pachymeninx (rather than the leptomeninx) have been much studied for their occurrence in migraine (21) and also observed in experimental autoimmune encephalitis (90, 91), trypanosomiasis (41), and infection by lymphocytic choriomeningitis virus (59). The immune cells may have arrived by extravasation from dural vessels (41, 92) or from the skull bone marrow (93) by way of the transcalvarial channels that contain veins (60, 94, 95). This recruitment of immune cells failed, however, to clear the pneumococci and was followed instead by sustained, albeit decreasing, densities of pneumoccci over days (Fig. S1). It therefore remains to be determined what conditions are permissive to the persistence of pneumoccci within the meninges, e.g., T regulatory-mediated mechanisms (96, 97). We found that 1-μm microspheres, as well as pneumococci (which have about the same diameter), translocate rapidly from the nasopharynx to the pachymeningeal compartment of the dorsal meninges. In the case of microspheres, this compartment was further distinguished from the subarachnoid space by injecting microspheres of a different color in the cisterna magna; it is known that from there, material is carried by CSF to the dorsal subarachnoid space (41, 49, 50) (Fig. 3). Furthermore, both microspheres and pneumococci appear to pass through the cribriform plate (Fig. 6C and D). Hence, we hypothesize that they translocate along the same pathway.

A limitation of our study resides in the dose of pneumococci instilled to each animal. While intranasal instillation represents in itself an artificial mean of infection, though very commonly used in murine models of respiratory pathogens, the concentration of pneumococci used in our study is based on pilot studies showing that a dose of 10^8^ pneumococci per mouse (in a volume of 10 μL) was necessary to produce consistent translocation of viable bacteria to the meninges. While a high dose of 10^8^ CFU may not be physiologically relevant, we estimate that a large percentage of pneumococci instilled to the mice will lyse prior to any dissemination. Further studies will be needed to draw a finer translocation time course of microspheres, pneumococcal cells, or pollutant particulates to investigate the effect of dose, shape, surface charges, and size on this rapid translocation.

At least five anatomical routes of transport through the foramina of the cribriform plate (12) have been proposed: transport within axons (anterograde along the olfactory axons [98, 99] or retrograde along the trigeminal axons [100]); transport within the olfactory nerve ensheathing cells (9, 101–103); transport along extracellular perineuralspaces of the nerves (9, 98, 104–106); transport in exiting lymph vessels (46, 107); and transport within or close to the periosteum (27). Objects as large as pneumococci or micrometer-sized microspheres diffuse much too slowly for diffusion to account for the rapid transport observed here, so either convection in a flowing fluid or some form of active transport is necessary. Axonal transport, typically 0.15 mm/min (108, 109) would take 33 minutes for a distance of 5 mm from nasopharynx into the cranial meninge and is therefore also too slow. Further arguments against such an intracellular route are that the olfactory axons are typically only 0.2 μm in diameter (110), much less than the diameter of pneumococci, and that sialic acid, a component of the extracellular glycocalyx of almost all cells (111), promotes translocation of pneumococci to the olfactory bulb (10, 11), which suggests that interaction with extracellular structures is important for the translocation process. As for convection, a puzzle is that numerous results show an efflux of fluid from cranium to nose, rather than an influx (112–115). The major conduits for efflux appear to be the spaces between the ensheathing cells that fasciculate the olfactory nerve (110, 116, 117) and the lymph vessels (46, 107). The former may drain the subarachnoid space (78, 116, 117) and the latter the pachymeninx (61, 118–120). Although there are reports of extracellular transport from the nasal mucosa toward the cribriform plate along the olfactory nerve (9, 78, 107, 121), a third extracellular route, described by Galeano et al. (27), along a space between the lamina propria and the turbinate bone, has the merits that it connects directly to the pachymeninx and has not been reported to carry an efflux. Of the known anatomical routes, this is therefore the most probable for the transport of pneumococci (Fig. 6F), for 1-μm microspheres and perhaps other particulate matter such as pollutants and drugs (28) targeting the CNS. Microspheres of diameters 5 and 10 μm were transported more slowly (Fig. 5), suggesting hindrance by the narrowness of spaces or by the presence of extracellular matrix.

Our results describe the early stages of pneumococcal entry into the central nervous system, upon intranasal challenge, and define the anatomical structures and fluid networks connecting the nasal cavity to the central nervous system and their barrier functions. By establishing that both pneumococci and microspheres translocate in minutes from the nasopharynx to the dorsal pachymeninx of mice, our data show for the first time the existence of a previously unrecognised inward flow of fluid through to the CNS. Should the CSF and/or brain parenchyma be subsequently invaded, this would mean that pneumococci (and perhaps any microparticles of similar size) would be capable of crossing the arachnoid barrier membrane. The exact mechanisms for this remain to be determined. Assuming similarities with animal models (122–125), our findings have significant implications for the diagnosis and clinical management of CNS infection in human patients. Further elucidation of the nasopharynx-to-meninges translocation route will have significant implications for the development of novel drug delivery systems to the brain and the etiology of brain damage caused by airborne particles such as pollutants.

## MATERIALS AND METHODS

### Ethics statement.

All animal experiments were conducted in accordance with the Animals (Scientific Procedures) Act 1986 and Amendment Regulations 2012 (ASPA 2012) and the care and maintenance guidelines of the Universities of Liverpool and Glasgow. All animal protocols were approved by the Local Animal Welfare and Ethics Committees under the UK Home Office Project License PB6DE83DA. In line with the 3Rs (Replacement, Reduction, and Refinement) principle, the number of animals was kept to a minimum and all surgery and intravital imaging were done under terminal anesthesia.

### Mice.

C57/BL6J female mice were obtained from Charles River Laboratories (Kent, UK) at 6 to 8 weeks old, maintained in an isolator, in a category II animal holding room and were allowed to acclimatize for at least 7 days before use. LysM^+^ cells were imaged in mice (a kind gift from Professor Sussan Nourshargh, Queen Mary University of London) in which the eGFP gene was knocked into the Lysozyme (Lys) M locus so that myelomonocytic cells were fluorescent, with neutrophils comprising the highest percentage of eGFP^hi^ cells (55). CD11c-eYFP mice are described in Lindquist et al. (65).

### Mouse models of Streptococcus pneumoniae infection.

S. pneumoniae serotype-2, strain D39 (NCTC 7466), were obtained from the National Collection of Type Culture, London, UK. Serotype-1 (sequence type 217) was a clinical isolate obtained from the cerebrospinal fluid of an adult male patient, archived at the Malawi-Liverpool-Wellcome Trust Clinical Research Centre, Blantyre, Malawi. The D39 strain was chosen for its value as a well-characterized benchmark isolate, while serotype 1 was used for its relevance as a high attack rate strain, i.e., very short periods of carriage with a high incidence of invasive disease (126). Both D39 and serotype 1 strains used in this study are known to be viable in blood up to at least 48 to 72 hours when administered intravenously (31, 127). Bacteria were streaked onto blood agar and grown overnight at 37°C, 5% CO_2_. Pneumococci were identified by the presence of a zone of hemolysis round each colony and a zone of inhibition round an optochin disc (128). A sweep of colonies was inoculated into brain heart infusion (BHI) broth (Thermo Fisher) and grown statically overnight at 37°C. The next day 750 μL of overnight growth was subcultured into BHI containing 20% (vol/vol) fetal calf serum (FCS) and grown statically for 4 to 6 hours until mid-log phase growth (ca. opitacal density at 500 nm [OD_500_] 0.8), at which point the broth was divided into 500-μL aliquots and stored at −80°C in BHI broth with FCS for no more than 1 month until use. Before use, two stock aliquots were thawed at room temperature, serially diluted from 10^−1^ to 10^−6^, and plated onto blood agar plates (129) to quantify CFU.

Mice were anesthetized with 2.5% isofluorane in oxygen and a 10-μL suspension containing 10^8^ CFU of serotype-2 (strain D39) or serotype 1 (ST217) was administered into the nostrils. Mice were returned to their cages and allowed to recover from anesthesia. Mice were then culled at times comprised between 15 minutes and 72 hours as well as up to 14 days postinfection. Health checks were performed at least three times a day on the infected animals; no changes in motor activity or other signs of disease were observed. To determine the viable counts (CFU) at the shortest time point possible, mice were killed by cervical dislocation immediately after recovery from anesthesia. The interval between the end of the intranasal administration and cardiac arrest was about 2 minutes 10 seconds. The tissue samples were dissected out 5 to 7 minutes later.

### Tissue collection for determination of CFU.

At least 100 μL of blood was taken by cardiac puncture, and in some cases, 2 to 5 μL of CSF was collected from the cisterna magna. Four different tissues were taken from each brain and immersed in 1.0 mL of sterile PBS. These were as follows: (i) the dorsal skull, excised with its adhering tissue, the cleavage plane is probably within the inner layers of the pachymeninx (16), so some pachymeningeal tissue may have been excluded: this tissue sample is called “skull/pachymeninx” in Fig. 1A: (ii) a layer of superficial tissue was sliced off from the dorsal cortex: these samples included the leptomeninx and probably inner layers of the pachymeninx, as well as parenchymal tissue (“cortex/leptomeninx”), (iii) the entire olfactory bulb, and (iv) the skull bone overlying the olfactory bulb, with its adherent meningeal tissue, labeled “skull/olfactory bulb.” The nasal cavity was exposed by removing the palate, and the nasal septum and associated nasal mucosa were harvested: “nasopharynx.” Tissue samples were homogenized using a T10 basic Ultra-Turrax homogenizer (IKA, Staufen, Germany) running at 30,000 rpm for 6 to 8 seconds at room temperature. One-hundred microliters of the homogenate was transferred to a well on a 96-well plate and 10-fold serial dilutions made in sterile PBS. Sixty-microliter aliquots were spotted on blood agar plates containing 10 μg/mL gentamicin. CSF samples were plated neat. Colonies were counted manually after overnight incubation under anaerobic conditions. To compare densities of CFU in pachymeningeal tissue scraped from the skull and (cortex + leptomeninx) samples, one volume of lysis buffer (125 mM Tris pH 6.8; 5 mM EDTA; 1% SDS; 10% glycerol) was added to one volume of undiluted homogenate, and protein content was assayed using a Pierce BCA Protein assay kit (Thermo Fisher) according to the manufacturer’s instructions.

### Fluorescence labeling of Streptococcus pneumoniae with CFSE.

Pneumococcus serotype 2, strain D39 were fluorescence labeled according to a previously described protocol (130). After growth to 0.5 OD_500_ in BHI growth medium at 37°C anaerobically, 1 mL of the suspension was transferred to a 1.5-mL tube and centrifuged at 4,000 × *g* for 5 min. The supernatant was discarded and the pellet resuspended in 1 mL of BHI containing 10 μM 5(6)-carboxyfluorescein diacetate *N*-succinimidyl ester (CFSE-SE; Sigma no. 21888). CFDA-SE does not affect the viability of pneumococci (130–132). The suspension was incubated on a rotating shaker at 37°C and 200 rpm for 45 minutes, in the dark, centrifuged at 12,000 × *g* for 3 minutes and washed three times with room-temperature PBS. The bacteria were resuspended at 10^8^ CFU/10 μL and stored on ice.

### Postmortem imaging of the meninges.

After the mouse was euthanized, the brain and meninges were perfused through the right cardiac atrium with either 50 mL PBS or with DiI-glucose solution according to a previously described protocol (42), followed by 50 mL of 4% (wt/vol) paraformaldehyde (PFA) solution, at 1.4 mL/min. The lower jaw and the scalp were removed to expose the dorsal and olfactory bulb areas of the skull. Either ventral brain tissue was removed from the cranium to leave about 0.5 mm soft tissue attached to the skull (the “brain-skull preparation”) and the meninges were imaged through the skull, or the brain parenchyma and the leptomeninx were removed to leave pachymeningeal tissue which was imaged from the internal face (40, 90, 91). The pieces of skull were mounted on a Petri-dish and imaged immediately. Z-stack images were obtained with a Zeiss LSM 880 two-photon microscope with femtosecond excitation at 840 nm with a ×10, N.A. 0.3 air or a ×20, N.A. 1.0 water immersion objective. CFSE was detected at 500 to 550 nm, Nile Red at 570 to 620 nm. Image stacks were 250 to 500 μm deep and comprised between 70 and 240 images with areas up to 425 × 425 μm.

### Baclight Red staining and LYVE-1 immunostaining of the skull whole mount.

Pneumococci were stained using BacLight Red stain (Thermo Fisher), a general cytoplasmic stain (44) following the manufacturer’s instructions i.e., 1 μL of a 100 μM DMSO working solution of the BacLight Red bacterial stain was added to 1 mL of bacterial suspension grown to mid-log phase, followed by two washes in PBS (0.1 M, pH 7.4) and resuspended in 100 μL of PBS. Ten microliters per mouse of the BacLight Red-stained Sp suspension was administered intranasally. At 15 minutes postadministration, mice were sacrificed by CO_2_ asphyxia and perfused with heparin-supplemented PBS solution, followed by 4% paraformaldehyde. The dorsal skull was carefully detached from the brain and trimmed to an area comprising the parietal and frontal bones together with attached meningeal tissue. The resulting tissue was stained using anti-mouse LYVE-1 monoclonal antibody (Thermo Fisher; ALY7-eFluor 450) diluted at 1:200 in PBS, and mounted in a Petri dish with intracranial face upwards for subsequent imaging. The mounted sample was imaged with a Zeiss LSM 880 confocal microscope with excitation set at 561 nm (for Baclight Red) and 405 nm (for LYVE-1). Images were acquired through a ×10, N.A. 0.3 air immersion objective. Baclight Red was detected at 571 to 664 nm while LYVE-1-eFluor 450 was detected at 416 to 538 nm.

### Intravital two-photon microscopy through the thinned skull.

The microscope and methods were essentially as previously described (20). Briefly, the mouse was maintained under isofluorane anesthesia, adjusted as necessary to suppress the withdrawal reflex, and core temperature was maintained at 37°C with a heating mat. The dorsal skull was exposed and a steel plate with a hole 5 mm in diameter was glued to the skull, usually with its center about 2 mm caudal to bregma and 2 mm lateral, and held in clamps. In some cases, the mouse was injected through a tail vein with a blood marker such as 70-kDa dextran-rhodamine and also furamidine, a nuclear dye that extravasates in the pachymeninx (41, 133). The skull within the hole in the plate was superfused with Tris-buffered saline and thinned with a dental drill. The mouse with attached plumbing was transferred to the stage of an upright two-photon microscope (Zeiss LSM7 MP) controlled by Zen software. The excitation source was a tunable femtosecond laser (Coherent Chameleon Ultra II). This was either used at a wavelength of up to 950 nm or set at up to 880 nm and used to drive an optical parametric oscillator (Coherent), which gave a second beam, typically set at 1,140 nm (for mKate). Images were acquired through a ×20, N.A. 1.0 water immersion objective (W Plan-Apochromat; Zeiss). Five detector channels were available to separate emission from different fluorophores and from second harmonic generation from bone and collagen. To follow leukocyte movement, z-stacks about 30 μm deep were collected at intervals of about 30 seconds.

### Image analysis.

Two-photon z-stacks and videos were analyzed with Imaris 9.5 (Bitplane) and Fiji (NIH Image) software packages. To separate neutrophils from other, less bright, cells in LysM^+^-eGFP mice, each movie was normalized to the same mean brightness and contrast was set manually against images obtained from an uninfected mouse. Cells were further selected for xy-diameters 12 μm or greater and identified as neutrophils. The approximate mean speeds were calculated from their positions in sequential 30 μm z-stacks obtained at the minimum repetition interval, typically 30 seconds. The number of LysM^+^ within each z-stack were quantified using the “spots” function. Values were then converted to the number of cells per mm^2^ according to the size of the imaging area. In order to enhance signal inside the region of interest (below the skull), the surface rendering of the skull as visualized by the SHG was generated and any signal found above this generated surface (outside the skull), was set to 0. To measure the distances from the skull to fluorescent pneumococci (or microspheres) in the 3D reconstructions, the distance measurement function of Imaris was used to calculate the shortest distance from the center of the positive signal to the surface rendering of the skull.

### Flow cytometric analysis.

Groups of C57BL/6J female mice (*n* = 5/time point) were instilled with S. pneumoniae D39 and euthanized at times ranging from 1 to 18 hours postinstillation. Pachymeningeal tissue was scraped from the calvaria, gently crushed, and passed through a cell strainer to produce a single cell suspension in Dulbecco’s phosphate-buffered saline (Thermo Fisher). Cells were counted and stained with anti-mouse antibodies to CD45 (clone 30-F11; BD Biosciences), CD4 (clone RM4-5; Biolegend), CD11b (clone M1/70; eBioscience), CD11c (clone N418; eBioscience), and LySM D1 (clone G3; Santa Cruz Biotechnology) or Ly6G (clone 1A8; Biolegend) in the presence of anti-CD16/32 Fc-receptor block (BD Biosciences). Events were acquired using a FACS Canto II (BD Biosciences) flow cytometer (Fig. S4).

10.1128/mbio.01024-22.3 FIG S3 Confocal images of mouse whole skull mount with transmitted light. S. pneumoniae stained with BacLight Red were instilled in the nose. At 15 minutes postadministration, mice were perfused transcardially with PBS followed by fixing solution (4% PFA). Dorsal skulls were mounted intracranial face upwards and stained with anti-LYVE1 antibody and imaged on the skull bone-oriented surface (from the exposed pachymeninx) with excitation at 561 nm. Representative maximum intensity z-projections are shown for an Sp-infected (A) and an uninfected (B) mouse, with transmitted light included (z = 239.95 μm). Download FIG S3, TIF file, 1.9 MB. Copyright © 2022 Audshasai et al. 2022 Audshasai et al. https://creativecommons.org/licenses/by/4.0/ This content is distributed under the terms of the Creative Commons Attribution 4.0 International license.

10.1128/mbio.01024-22.4 FIG S4 Immune cell FACS gating strategy. Flow cytometric discrimination of Ly6G^+^, LysM^+^, and CD11c^+^ cells by surface marker expression. Representative FACS plots, which detail our 6-color flow cytometry gating strategy of single cell suspensions obtained from the mouse pachymeningeal tissues from an Sp-infected mouse at 10 hours postinfection. Neutrophils were defined as CD45^+^ CD4neg CD11bneg/dim Ly6G^+^, and myelomonocytic cells were defined as CD45^+^ CD4neg CD11bneg/dim LysM^+^. Dendritic cells were defined as CD45^+^ CD4neg CD11bhigh CD11c^+^. Download FIG S4, EPS file, 1.9 MB. Copyright © 2022 Audshasai et al. 2022 Audshasai et al. https://creativecommons.org/licenses/by/4.0/ This content is distributed under the terms of the Creative Commons Attribution 4.0 International license.

### Intranasal administration of microspheres.

Fluorescent polystyrene microspheres of three nominal diameters were used: yellow-green (505/515 nm; Thermo Fisher F13081; 4 × 10^10^ microspheres/mL) and Nile Red (Thermo Fisher F8819; 4 × 10^10^ microspheres/mL) nominally 1 μm with carboxylate-modified surface, Nile Red nominally 5 μm (5 to 7.9 μm) (Spherotech FP-6056-2; 1.5 × 10^8^/mL) and Nile Red nominally 10 μm (10 to 14 μm) (Spherotech FH-10056-2; 10^7^ microspheres/mL) with unmodified surfaces. For imaging and flow cytometry on meningeal tissue, suspensions containing approximately 10^7^ microspheres/mL were prepared and a volume of 10 μL (containing 10^5^ microspheres) was applied intranasally to each of five mice for each diameter and the mice were culled by CO_2_ asphyxia 30 minutes later. In experiments designed to label the leptomeninx, microspheres were injected in the cisterna magna using a 34-G 10-μL microsyringe (Hamilton). All of these experiments involving microspheres were conducted without any prior or subsequent coadministration with Sp.

Microspheres were imaged *ex vivo* either through the skull and into the meninges overlying the olfactory bulb and the dorsal cortex (the brain-skull preparation) or from the intracranial face of the skull with its attached pachinmeningeal tissue (the “amaguri” preparation). Excitation was at 840 nm, which produced two-photon excitation of the fluorophores and SHG (in blue). For flow cytometry, pachymeningeal tissue scraped from the dorsal was collected in PBS and passed through a cell strainer. A BD Canto II flow cytometer detected the YFP- and Nile Red-labeled microspheres using the FITC and PerCP Cy5.5 channels, respectively.

### Statistical analysis.

For comparison of multiple groups, the statistical significance of endpoints was evaluated by one-way ANOVA followed by Tukey’s multiple comparisons *post hoc* test. For comparison of two groups, the unpaired two-tailed Student's *t* test was used. Data are presented as means ± SEM in bar graphs. Statistical significance is reported as *, *P* < 0.05, **, *P* < 0.01, ****, *P* < 0.0001. All statistical analyses were performed with Prism software (version 8.0, GraphPad Software).
