# Supplementary figures and images for: Streptococcus pneumoniae Rapidly Translocate from the Nasopharynx through the Cribriform Plate to Invade the Outer Meninges
Source: mBio. 2022 Aug 4;13(4):e01024-22. doi: 10.1128/mbio.01024-22 (PMC9426477; doi:10.1128/mbio.01024-22)

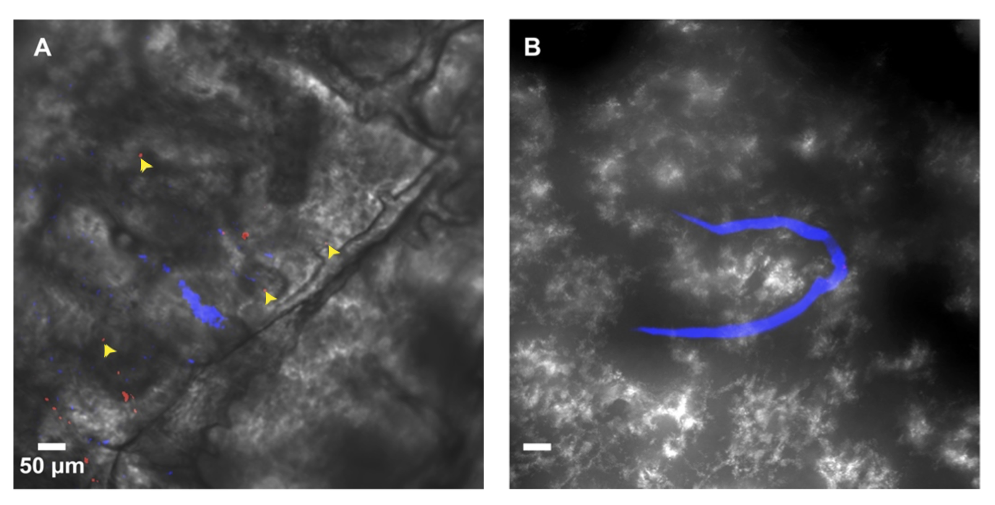

Supplement: FIG S3 [file mbio.01024-22-s0003.tif]
